# Multi-Objective Topology Optimization of a Broadband Piezoelectric Energy Harvester

**DOI:** 10.3390/mi14020332

**Published:** 2023-01-27

**Authors:** Siyang Hu, Ulrike Fitzer, Khai Chau Nguyen, Dennis Hohlfeld, Jan G. Korvink, Tamara Bechtold

**Affiliations:** 1 Department of Engineering, Jade University of Applied Sciences, Friedrich-Paffrath-Str. 101, 26389 Wilhelmshaven, Germany; 2 Institute for Electronic Appliances and Circuits, Faculty of Computer Science and Electrical Engineering, University of Rostock, Albert-Einstein-Str. 2, 18059 Rostock, Germany; 3 Institute of Microstructure Technology, Karlsruhe Institute of Technology, Hermann-von-Helmholtz-Platz 1, 76344 Eggenstein-Leopoldshafen, Germany

**Keywords:** topology optimization, broadband energy harvesting, piezoelectric energy harvesting

## Abstract

In recent years, topology optimization has proved itself to be state of the art in the design of mechanical structures. At the same time, energy harvesting has gained a lot of attention in research and industry. In this work, we present a novel topology optimization of a multi-resonant piezoelectric energy-harvester device. The goal is to develop a broadband design that can generate constant power output over a range of frequencies, thus enabling reliable operation under changing environmental conditions. To achieve this goal, topology optimization is implemented with a combined-objective function, which tackles both the frequency requirement and the power-output characteristic. The optimization suggests a promising design, with satisfactory frequency characteristics.

## 1. Introduction

Energy harvesting devices convert free ambient energy into usable electrical energy. This energy can then be used as an additional power source for wireless autonomous devices, e.g., inside a wireless sensor network. These devices are often deployed under harsh environmental conditions, causing them to be not easily accessible [1]. There are different sources of ambient energy available for energy harvesting, such as solar, wind, and mechanical energy. In our works, we focus on energy harvesting from mechanical vibrations that occur due to the motion of rotatory machines, e.g., in an industrial setting or in automotive. Different techniques are applicable for vibrational energy harvesting: electromagnetic, electrostatic, magnetostrictive, and piezoelectric [2]. Of all these, piezoelectric energy harvesters (PEH) are the most common type of vibration energy harvesters, due to their high achievable power density, scalability, and simplistic geometry [3,4,5,6]. The simplest design consists of a flexible beam structure that carries piezoelectric material, which transforms vibration energy into usable electric power. Typically, a proof mass is added, in order to achieve the desired resonant frequency for a given application. However, very often the ”harvesting frequency” changes over time, due to, e.g., changes in environmental conditions (temperature and humidity) or due to the aging of the vibration source. In such case, a simple PEH fails, as the structure represents a single mass–spring–damper system and, therefore, only exhibits a single dominant resonant mode.

An adaptation or tuning of the PEH’s resonant frequency is considered one of many broadband energy-harvesting techniques applied to avoid a mismatch of excitation frequency and the PEH’s resonant frequency. Comprehensive reviews on the topic of broadband piezoelectric energy harvesting can be found in [7,8]. In this work, we consider a so-called multi-modal broadband energy-harvesting approach. Multi-modal energy harvesting requires a device that exhibits multiple resonant frequencies that, therefore, can cover a larger frequency bandwidth. In [9], a bi-directional, U-shaped structure with cross-connected beams was introduced that can capture vibration in 3D space. In [10,11], ”star”- and a ‘’pizza”-shaped arrays were proposed and, in [12], a trapezoidal geometry and optimal segmentation for higher modes were presented. Most of these recent designs are based on combining multiple simple beam PEHs with different resonant frequencies into an array, which is often referred to as an ”in parallel” configuration. In our case, we focus on serial designs, i.e., cascading simple beam PEHs instead of arranging them into an array [13]. In [14], we showed that cascading/serial designs significantly outperform array configurations and confirmed previously presented results from, e.g., [15,16].

We refer to our compact dual-frequency resonator as the folded-beam design. Its design goal is to have the first two resonant frequencies with similar power amplitudes in close proximity. Therefore, it can provide a broad usable frequency range for energy harvesting. This design approach is our key differentiation from other authors. A major challenge of our design is to account for unwanted interactions between the two modes, such as switching or merging. That is why manual design approaches become unfeasible and mathematical optimization approaches are to be favored.

The authors of [17] discuss various optimization algorithms with regard to their respective suitability for finding the suitable geometric dimensions of PEHs. More device-specific optimizations are presented in [18], where multiple topologies of a tip-excited single beam PEH are parametrically optimized over a power spectrum from 0 Hz to 800 Hz. In [19], the parameter optimization of a magnetic coupled PEH was performed, optimizing the spacing of two coupled resonators for different magnetic flux densities. In [20], stack PEH for automotive is optimized, with regard to parameters, such as the number of stack layers or height to cross-section ratio. For the folded-beam PEH, we implemented a two-stage global parametric design optimization and proposed novel geometries in [14,21,22]. Parameter optimizations, however, are strongly limited, as the topology of the final design is already determined by the initial design. For example, the final design cannot be star-shaped if the initial design was rectangular. Hence, in this work, we explore topology optimization (TO) to gain more design freedom.

The methodology of TO was introduced in [23] and has become very popular across all fields of engineering. The TO methodology is designed to find an optimal layout of a structure given the available design space, applied loads, support conditions, and constraints such as maximum weight. This is achieved by reformulating the classical optimization problem with a set of distributed functions on a fixed domain [24]. In most applications, this reformulation results in an optimal material distribution problem on a finite-element grid. The most common TO application is weight or compliance minimization. However, recent works show that TO is also considered for thermal applications [25], where a cold plate for a lithium-ion battery was optimized, or for optical applications [26], where the desired band gap characteristics for phononic crystals were obtained.

The first studies of TO on the eigenfrequency can be found in [27], where the support of a plate-like structure was optimized. Later, in [28,29], the modified optimality criteria and the mean frequency goal function were introduced, which significantly improved the performance of TO for dynamical eigenfrequency problems. In [30], we introduced a dynamic compliance-based optimization approach for resonant structures that uses the compliance goal function to have more control over the modal shape and for a more stable optimization process. As for applications, in [31], a 2D in-plane single mass MEMS gyroscopes were optimized using TO, which was later extended to a tuning fork resonator, i.e., a two mass configuration, in [32]. In [33], a composite material PEH was proposed as a result of a multi-material TO. More recently, the same authors proposed plate-type PEH designs [34], maximizing open-circuit voltage by topology optimizing the through-thickness of piezoelectric material distribution.

In this work, we present the TO of a multi-modal PEH and propose a novel folded-beam design that fulfills its unique frequency requirements. The TO procedure implements a multi-objective goal function to tackle both the frequency and the power amplitude requirements. The achieved optimal design is characterized in a simulation. The manuscript is structured as follows: the introduction of the folded-beam PEH is provided in Section 2; in Section 3, we introduce our TO approach and establish the optimization setup; and, in Section 4 we present the optimization results and discuss them in Section 5. Section 6 concludes this work and provides an outlook to future research.

## 2. Definition and Modeling of Multi-Modal Piezoelectric Energy Harvester

Piezoelectric materials can convert mechanical vibration into electrical energy using the piezoelectric effect. The corresponding governing equation is usually provided in coupled strain-charge form [35]:

(1) $$\begin{array}{cc} S & =sT+\sigma^{T}E, \end{array}$$

(2) $$\begin{array}{cc} D & =\sigma T+\epsilon E, \end{array}$$

where $S\in R^{6\times 1}$ and $T\in R^{6\times 1}$ are the stress and strain tensors, respectively, and D is the electric displacement vector. $\sigma \in R^{3\times 6}$$s\in R^{6\times 6}$ denotes the compliance tensor measured with a constant electric field E and $\epsilon \in R^{3\times 3}$ denotes the permittivity tensor at constant strain. σ is the piezoelectric tensor. As most piezoelectric materials are classified as ceramics, they are extremely brittle and require an elastic support structure for this designed use case. Therefore, a PEH is typically composed of an elastic mechanical resonator that carries piezoelectric material, which is attached to the areas that are exposed to mechanical stress or strain. The design of a PEH mostly focuses on the geometry of the mechanical resonator in order to achieve desired characteristics.

### 2.1. Finite Element Modeling of Piezoelectric Energy Harvester

The finite element discretization of the governing equation results in a second-order system of dynamic algebraic equations:

(3) $$\begin{array}{c} \Sigma =\left\{\begin{array}{c} \underset{=:M}{\underbrace{\left[\begin{array}{cc} M_{m} & 0\\ 0 & 0 \end{array}\right]}}\left[\begin{array}{c} {\ddot{x}}_{m}\\ {\ddot{x}}_{\mathrm{el}} \end{array}\right]+D\left[\begin{array}{c} {\dot{x}}_{m}\\ {\dot{x}}_{\mathrm{el}} \end{array}\right]+\underset{=:K}{\underbrace{\left[\begin{array}{cc} K_{m} & K_{m,\mathrm{el}}\\ K_{m,\mathrm{el}}^{T} & K_{\mathrm{el}} \end{array}\right]}}\left[\begin{array}{c} x_{m}\\ x_{\mathrm{el}} \end{array}\right]=\left[\begin{array}{c} b\\ 0 \end{array}\right]u\\ y=C\left[\begin{array}{c} x_{m}\\ x_{\mathrm{el}} \end{array}\right] \end{array}\right., \end{array}$$

where $x_{m}$ are the nodal displacements and $x_{\mathrm{el}}$ the nodal electrical potentials. $M_{m}$ and $K_{m}$ denote the mechanical mass and stiffness matrices, respectively. $K_{\mathrm{el}}$ is the dielectric matrix and $K_{m,\mathrm{el}}$ describes the piezoelectric coupling. The input *u* acts on the system via the input vector b and C gathers the user-defined outputs of the system y. Finally, D=αM+βK defines the Rayleigh damping for the system.

### 2.2. Folded-Beam Resonator

**Figure 1:**
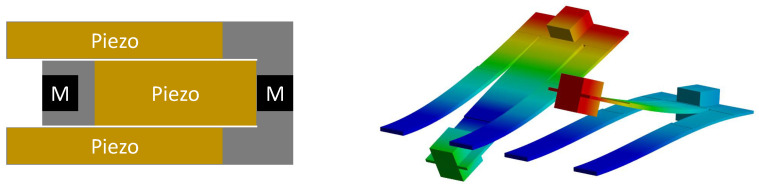
Folded-beam design introduced in [13]. The geometry is shown on the left. It is composed of a steel structure (gray), two tip masses (black), and three piezoelectric patches (mustard). The first two mode shapes of the structure are shown on the right.

The folded-beam design was introduced in [13]. Its geometry equals two cascading simple beam harvesters, with the second beam folded toward the fixed end of the first beam instead of further extending it. The initially proposed design is shown in Figure 1.

The design can be fabricated from a single sheet of steel. It includes piezoelectric films on both cantilever beams and permanent magnets at their free ends. These magnets can be used for bi-directional frequency tuning via external magnetic forces, which is, however, out of the scope of the current work. The folded-beam geometry is designed to have two fundamental modes, corresponding to the two respective beams, at specific frequencies in the range of from 50 Hz to 100 Hz.

## 3. Topology Optimization Procedure

In general, a TO problem is defined as follows:

(4) $$\begin{array}{c} \min_{\rho }g\left(\rho \right) \end{array}$$

subject to:

(5) $$\begin{array}{c} \Sigma :\left\{\begin{array}{c} M\left(\rho \right)\ddot{x}+D\left(\rho \right)\dot{x}+K\left(\rho \right)x=bu\\ y=Cx \end{array}\right. \end{array}$$

(6) $$\begin{array}{c} \sum_{e=1}^{n}\rho_{e}V_{e}-V^{*}\leq 0,\;\left(\rho ={\left[\rho_{e}\right]}_{e\in [1,n]},V^{*}=\alpha V_{0}\right), \end{array}$$

(7) $$\begin{array}{c} 0\leq \rho_{e}\leq 1, \end{array}$$

where the design parameter ρ is a vector containing all the elemental pseudo-densities $\rho_{e}\in \left[0,1\right]$. *g* is the objective function and Σ is the dynamical system resulting from finite element discretization introduced in (3). The (6) introduces a volume constraint for the optimization. The density-dependent system matrices are provided by the classical simple isotropic material model with penalization (SIMP model):

(8) $$M\left(\rho \right)=\sum_{e=1}^{n}\rho_{e}M_{e},\;K\left(\rho \right)=\sum_{e=1}^{n}\rho_{e}^{p}K_{e},$$

with $M_{e}$ and $K_{e}$, respectively, being the elemental mass and stiffness matrices of a full finite element.

### 3.1. Objective Function and Sensitivity Analysis

**Figure 2:**
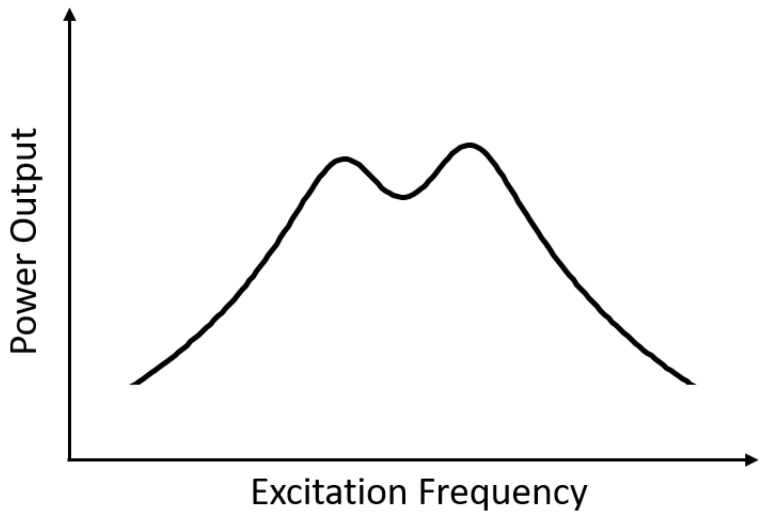
An ideal case for a frequency range of efficient operation, formed by two overlapping resonant modes (log scale).

The main design goal of the folded-beam PEH is to have the two fundamental modes inside a pre-defined small frequency range. In this way, their response peaks overlap and allow for efficient energy harvesting over the entire frequency range (cf. Figure 2). However, the modes are also required to generate similar levels of power output, in order to realize this usable frequency range, as if one of the modes has a significantly higher power output, the second mode may not even be observable. These two goals are taken into account via a combined-objective function that consists of two parts. The first part $g_{1}$ is the mean frequency objective function first introduced in [29], which has already been involved in our previous works [36]. It is defined as:

(9) $$g_{1}:={\left(\frac{1}{w_{1}+w_{2}}\left[w_{1}{\left(\lambda_{1}-\lambda_{1,\mathrm{goal}}\right)}^{2}+w_{2}{\left(\lambda_{2}-\lambda_{2,\mathrm{goal}}\right)}^{2}\right]\right)}^{\frac{1}{2}},$$

where $\lambda_{1},\lambda_{2}$ and $\lambda_{1,\mathrm{goal}},\lambda_{2,\mathrm{goal}}$ are the first two eigenvalues of the generalized eigenvalue problem corresponding to model (3) and their respective target values. Note that each of the eigenfrequencies of the strucure $f_{i}$ are connected to $\lambda_{i}$ as follows: $\lambda_{i}={\left(2\pi f_{i}\right)}^{2}$. The weighting factors $w_{1}$ and $w_{2}$ are chosen as $1/\lambda_{1,\mathrm{goal}}$ and $1/\lambda_{2,\mathrm{goal}}$, respectively. This nondimensionlizes the expression and ensures an equal convergence for all eigenvalues [29]. The second part of the objective function $g_{2}$ represents the relative difference of the electrical energy of the structure at both resonant modes $p_{el,1}$ and $p_{el,2}$, i.e.:

(10) $$g_{2}:={\left(\frac{2(p_{el,1}-p_{el,2})}{p_{el,1}+p_{el,2}}\right)}^{2}.$$

The dielectric energy of the structure [37,38] can be computed as:

(11) $$p_{el,i}=\frac{1}{2}\phi_{i,\mathrm{el}}^{T}K_{\mathrm{el}}\phi_{i,\mathrm{el}},$$

where $\phi_{i,\mathrm{el}}$ contains the electrical degrees of freedom of the *i*-th eigenvector. The partial objective functions are combined in a weighted sum:

(12) $$g=v_{1}g_{1}+v_{2}g_{2},$$

and, analogously, the sensitivity equals the weighted sum of both partial sensitivities:

(13) $$\frac{\partial g}{\partial \rho_{e}}=v_{1}\frac{\partial g_{1}}{\partial \rho_{e}}+v_{2}\frac{\partial g_{2}}{\partial \rho_{e}}.$$

The weighting factors $v_{1}$ and $v_{2}$ are chosen such that both sensitivities are in the same order of magnitude. The partial sensitivities can be computed as:

(14) $$\begin{array}{c} \frac{\partial g_{1}}{\partial \rho_{e}}=\frac{1}{\left(w_{1}+w_{2}\right)g_{1}}\left[w_{1}\left(\lambda_{1}-\lambda_{1,\mathrm{goal}}\right)\frac{\partial \lambda_{1}}{\partial \rho_{e}}+w_{2}\left(\lambda_{2}-\lambda_{2,\mathrm{goal}}\right)\frac{\partial \lambda_{2}}{\partial \rho_{e}}\right] \end{array}$$

for the first part, where $\frac{\partial \lambda_{i}}{\partial \rho_{e}}$ is obtained by (cf. Appendix A):

(15) $$\frac{\partial \lambda_{i}}{\partial \rho_{e}}=\phi_{i,e}^{T}\left(\frac{\partial K_{e}}{\partial \rho_{e}}-\lambda_{i}\frac{\partial M_{e}}{\partial \rho_{e}}\right)\phi_{i,e}.$$

The second part of the sensitivity is obtained using the quotient rule:

(16) $$\frac{\partial g_{2}}{\partial \rho_{e}}=8\sqrt{g_{2}}\frac{\left(\frac{\partial p_{el,1}}{\partial \rho_{e}}-\frac{\partial p_{el,2}}{\partial \rho_{e}}\right)\left(p_{el,1}+p_{el,2}\right)-\left(p_{el,1}-p_{el,2}\right)\left(\frac{\partial p_{el,1}}{\partial \rho_{e}}+\frac{\partial p_{el,2}}{\partial \rho_{e}}\right)}{{\left(p_{el,1}+p_{el,2}\right)}^{2}},$$

where $\frac{\partial p_{el,i}}{\partial \rho_{e}}$ are, again, computed via the adjoint method (cf. Appendix B):

(17) $$\frac{\partial p_{el,i}}{\partial \rho_{e}}=\mu_{i,m}^{T}\left(\frac{\partial K_{m}}{\partial \rho_{e}}-\lambda_{i}\frac{\partial M_{m}}{\partial \rho_{e}}\right)\phi_{i,m},$$

and $\mu_{i,m}$ is the mechanical part of the adjoint state vector that solves the adjoint problem:

(18) $$\left[\begin{array}{cc} K_{m}-\lambda_{i}M_{m} & K_{m,\mathrm{el}}\\ K_{m,\mathrm{el}}^{T} & K_{\mathrm{el}} \end{array}\right]\left[\begin{array}{c} \mu_{i,m}\\ \mu_{i,\mathrm{el}} \end{array}\right]=\left[\begin{array}{c} 0\\ \frac{1}{2}K_{\mathrm{el}}\phi_{i,\mathrm{el}} \end{array}\right].$$

### 3.2. Filters

**Figure 3:**
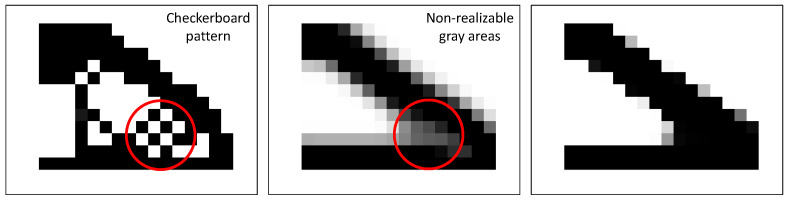
Filter techniques in TO. **Left**: A TO-obtained structure without any filter. **Center**: Same optimization with a density filter. **Right**: Same optimization with both filters active.

Having elemental pseudo-density as its design variable, TO suffers from a number of issues. Firstly, the solution depends on the discretization of the design domain. Finer meshes often generate thin details that cannot be obtained by coarser meshes and would therefore converge to a completely different topology. Secondly, TO on a coarse mesh often converges to designs suffering from the well-known checkerboard problem [39,40]. These problems can be tackled by applying appropriate filter techniques [24,41], these filters themselves, however, introduce a third issue. The aforementioned filtering techniques regularize elemental density based on a weighted average of the elemental density of adjacent elements and, therefore, introduce blurred, i.e., gray areas with intermediate elemental densities, which are not manufacturable. We have experienced this issue extensively in our previous works [30,36]. In order to resolve this issue and to obtain manufacturable designs, we have implemented a Heaviside filter utilizing the threshold projection suggested in [42]. Figure 3 shows both filters and their effects on the same optimization process.

To summarize, we have implemented both the density filter and the threshold projection in our optimization to achieve a manufacturable design. First, we apply a density filter on the candidate design encoded in ρ, which is suggested by the optimizer. Subsequently, the density-filtered design $\tilde{\rho }$ is projected onto a pre-defined threshold using the Heaviside filter, providing us with the final design $\hat{\tilde{\rho }}$. Both filters will be introduced in detail in the following subsections.

#### 3.2.1. Density Filter

Let $\rho_{i}$ be the elemental pseudo-density of the *i*-th element computed by the optimizer. The density filter replaces this value with ${\tilde{\rho }}_{i}$ by performing a weighted average of its adjacent elemental densities:

(19) $${\tilde{\rho }}_{i}=\frac{\sum_{j\in N_{e,i}}w\left(r_{j}\right)\rho_{j}}{\sum_{j\in N_{e,i}}w\left(r_{j}\right)},\;w\left(r_{j}\right)=R_{\min}-\left|r_{i}-r_{j}\right|,$$

where $N_{e,i}$ is the set of adjacent elements in the neighborhood of element *i* and w(r) is the weight function based on a user-specified filter radius $R_{\min}$ and the Euclidean distance between the elements *i* and *j* ($r_{i},r_{j}$ denotes the coordinates of the center point of the respective element).

Accordingly, the sensitivity of the density filter (19) with reference to the design variable is provided by:

(20) $$\frac{\partial {\tilde{\rho }}_{i}}{\partial \rho_{j}}=\frac{w(r_{j})}{\sum_{j\in N_{e,i}}w\left(r_{j}\right)}.$$

Equation (20) needs to be added to the sensitivity of the goal function according to the chain rule.

#### 3.2.2. Threshold Projection

**Figure 4:**
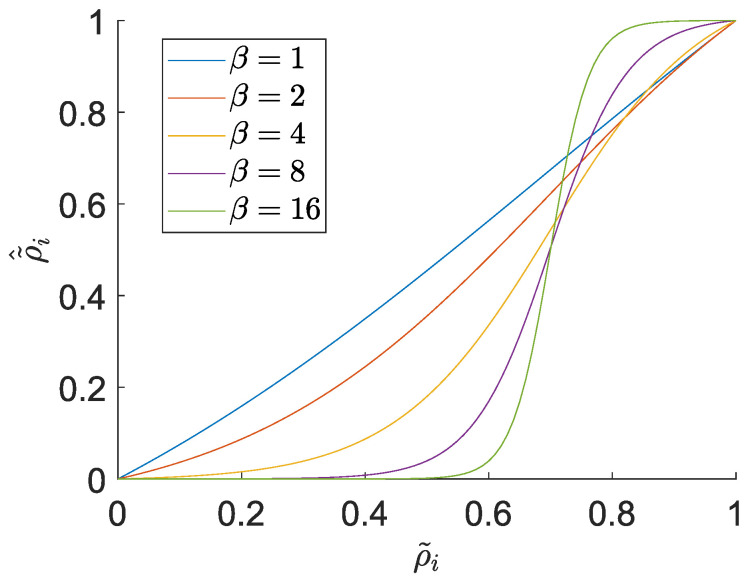
Threshold projection (21) for threshold η=0.7 and different β values.

The threshold projection sets all the density values above a user-defined threshold to 1 and all the values below this threshold to 0. There are several versions of this filter. In [42], the filter is constructed as follows:

(21) $${\hat{\tilde{\rho }}}_{e}=\frac{\tanh\left(\beta \eta \right)+\tanh(\beta \left({\tilde{\rho }}_{e}-\eta \right))}{\tanh(\beta \eta )+\tanh(\beta (1-\eta ))},$$

where η is the projection threshold and β is a projection parameter tuning the ”sharpness” of the projection (cf. Figure 4).

The corresponding elemental correction term for the sensitivity is provided by:

(22) $$\frac{\partial {\hat{\tilde{\rho }}}_{e}}{\partial {\tilde{\rho }}_{e}}=\frac{1}{\beta \cosh^{2}\left(\beta \left({\tilde{\rho }}_{e}-\eta \right)\right)\left[\tanh(\beta \eta )+\tanh(\beta (1-\eta ))\right]}.$$

## 4. Optimization Results

**Figure 5:**
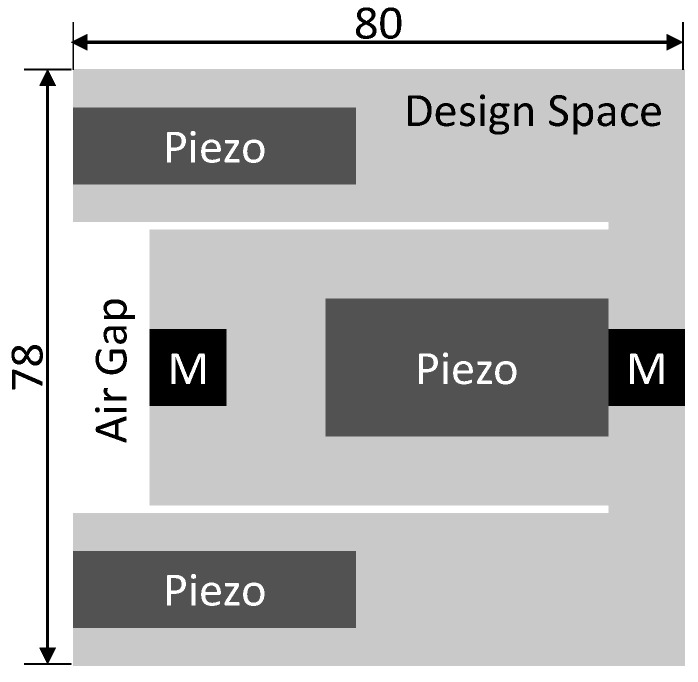
Design space of the problem. The areas beneath the piezo (-electric) patches and the masses M are excluded from the design space.

The optimization is initialized with a design space that measures 80×78 ${\mathrm{mm}}^{2}$. The design space is discretized with 1×1×1 ${\mathrm{mm}}^{3}$ linear cubic finite elements. From this design space, we exclude regions reserved for the application of commercially available piezoelectric patches as well as pre-defined gaps between the inner and outer beam, as well as between the inner beam and the fixed end. Figure 5 depicts the setting for the optimization. The design space is marked light gray. Both masses (M) are modeled as point masses, as we found this approximation to be sufficiently accurate for our application. Finally, the piezoelectric patches are modeled as a secondary layer on top of the design space using the material parameters provided in [43]. The material parameters are presented in Table 1.

**Figure 6:**
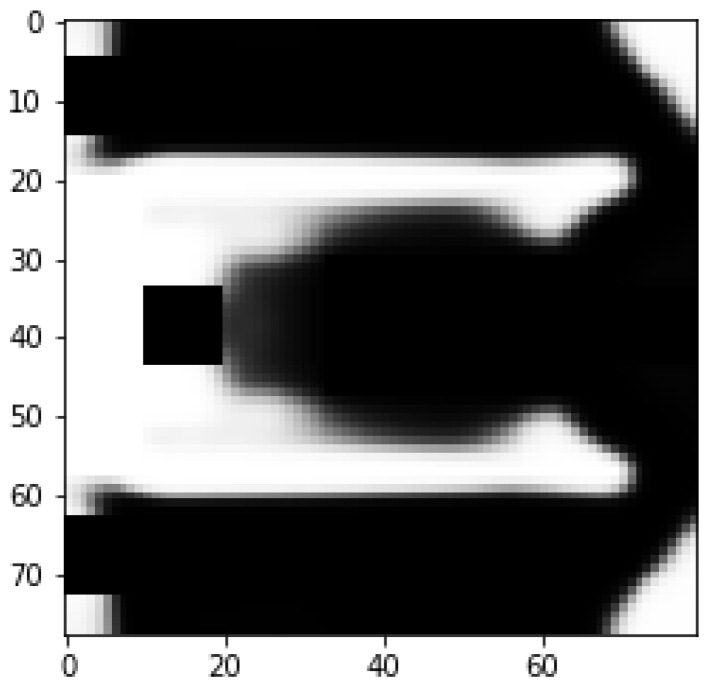
Optimized geometry. The gray level indicates the value of $\rho_{e}$ for each element.

For this contribution, the target values for the structure’s resonant frequencies are set to $f_{1}=74\;\mathrm{Hz}$ and $f_{2}=76\;\mathrm{Hz}$. Furthermore, the penalty factor is set to p=2 and the volume fraction to α=0.7. At the beginning of the optimization, β=1 and this parameter is subsequently increased during optimization until β=8. The optimization is stopped either if the maximum change of elemental density is less than 1% or when the number of iterations reaches 100. The resulting geometry is presented in Figure 6. The structure’s resonant frequencies are $f_{1}=73.59\;\mathrm{Hz}$ and $f_{2}=74.48\;\mathrm{Hz}$. The relative difference in dielectric energy between these resonant modes is $\sqrt{g_{2}}=15.03\%$.

**Figure 7:**
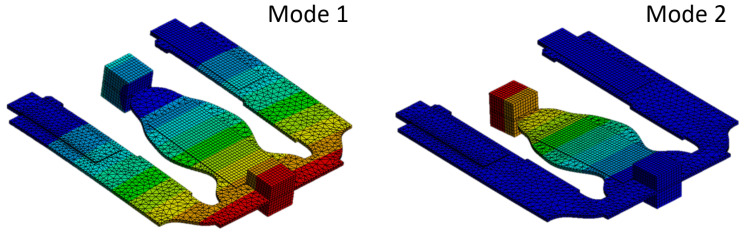
Mode shapes of the first two resonant modes of the optimal structure after the conversion to a manufacturable design value at $f_{1}=74.573\;\mathrm{Hz}$ and $f_{2}=79.054\;\mathrm{Hz}$.

**Figure 8:**
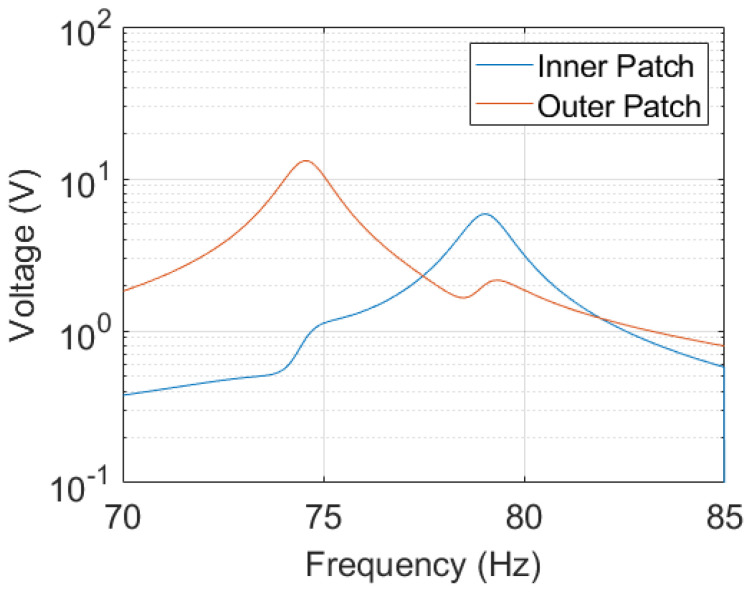
Open- circuit voltage output of the structure obtained through harmonic analysis. The structure is excited with 0.2 g at its fixed end. The damping is set to 0.008.

**Figure 9:**
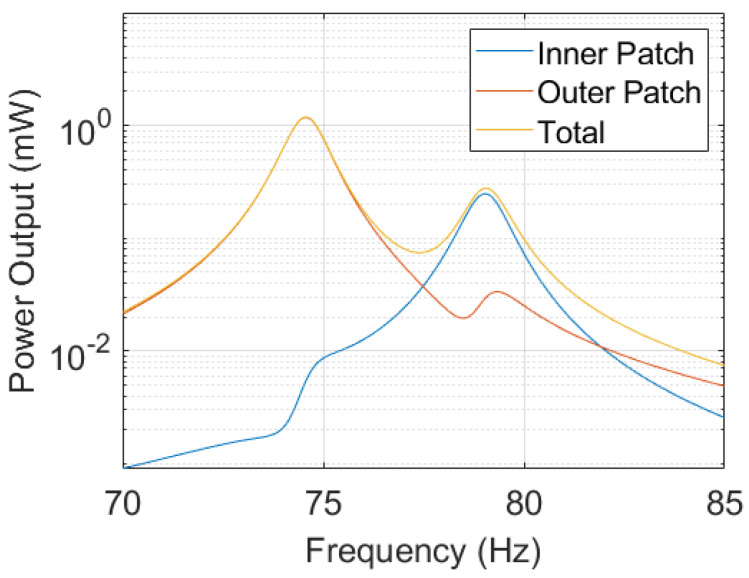
Maximum power output of the structure at optimal load value computed from values presented in Figure 8. Both outer patches are connected in parallel. The total power output is computed as the sum of both patches.

To achieve a manufacturable design, the final geometry is cut-off at $\rho_{e}=\alpha$ (volume fraction), i.e., elements with $\rho_{e}>\alpha$ are considered full and those with $\rho_{e}\leq \alpha$ are considered void. The boundary of the geometry is smoothed as the final step of this conversion. Figure 7 shows the mode shapes of the first two resonant modes of the structure after conversion. They value at $f_{1}=74.573\;\mathrm{Hz}$ and $f_{2}=79.054\;\mathrm{Hz}$, respectively. The corresponding open-circuit voltage and maximum power outputs are presented in Figure 8 and Figure 9. For the computation of the electrical power output, we assumed optimally matched load resistances at each frequency. Note that both outer patches are connected in parallel.

## 5. Discussion

As presented in Section 4, the intended objectives are not achieved by the optimization. This is to be expected, as the optimization result can only partially fulfill both objective contributions ($g_{1}$ and $g_{2}$). Furthermore, we observe a change in the resonant frequency $f_{2}$ after deriving a manufacturable geometry from the optimization result. However, both values are still acceptable for our intended application.

After analyzing the power output of the manufacturable geometry using a harmonic analysis (Figure 9), we observe a bigger relative difference in the power amplitudes at the two resonant frequencies compared to the optimization process. During the optimization, the structure’s dielectric energy has been used as a power potential indicator (as implemented in $g_{2}$). After optimization, the relative difference of the dielectric energy $\sqrt{g_{2}}$ at both resonant frequencies was minimized to 15%, whereas the relative difference in the power output values to 124%. There are several factors contributing to this discrepancy. Firstly, we use the eigenvectors for the evaluation of the dielectric energy of the system in (11). This is computationally efficient, as the mode shapes, i.e., the eigenvectors, are directly obtained during the computation of the resonant frequencies. By definition, the nodal displacements and potentials in the eigenvectors represent the mode shape and cannot be evaluated quantitatively. Hence, proper scaling of the eigenvectors would improve the accuracy of the indicator. Such an improvement can be implemented by a harmonic analysis within the optimization loop, which motivates the following discussion.

Our experimental observations revealed that the damping of a multi-resonant structure is mode-specific and generally dependent on frequency. It was not possible to determine a single damping ratio, which applies to the whole frequency range under investigation. Common practice is to adjust the damping model, such that the simulation results match the experimental findings. Due to the unavailability of a reliable extent of damping, we have to state that the additional computational effort for the calculating power output from a harmonic analysis is of limited use. Thereby, we favored the use of dielectric energies as obtained from the eigenvectors for power estimation. Only experimental characterization can provide mode-specific damping information and resulting power output.

## 6. Conclusions and Outlook

In this work, we performed a TO of a multi-resonant PEH. For the optimization, we proposed a novel combined-objective function with the following requirements: (1) the final structure should have its first two resonant frequencies at $f_{1}=74\;\mathrm{Hz}$ and $f_{2}=76\;\mathrm{Hz}$ and (2) the relative difference in the power output value corresponding to the two modes should be minimal. To obtain a manufacturable design, a regularization filter for the elemental material density has been implemented. The optimization has converged to a promising structure that fulfills the requirements. In post-processing, the optimal result is converted into a manufacturable geometry. The subsequent finite element simulation of this manufacturable design shows satisfactory results for the structure’s resonant frequencies. The corresponding power output amplitudes can be improved. However, these values may not be a true representation of the device’s real-world performance as, in the course of simulations, constant damping was assumed. This assumption is not realistic but is a common practice, since accurate frequency-dependent damping models can only be obtained experimentally. Therefore, only an experimental characterization can determine the actual power output level of the device.

As a next step, we will fabricate the optimized design obtained from this work and perform experimental characterizations for further validation of the design approach. Furthermore, we will implement a harmonic analysis of the power output values within the optimization loop for a more accurate estimation of the output power. The additional computational effort will be minimized by the state-of-the-art model order-reduction techniques.

## Figures and Tables

**Table 1:** Mechanical properties of commercial MFC piezoelectric patches [43].

Density (kg/m${}^{3}$)		Piezoelectric Constants (C/m${}^{2}$)	
ρ	4700	$e_{31}$	−2.227
**Young’s Modulus (GPa)**		$e_{32}$	−0.671
$E_{1}$	45.21	$e_{33}$	16.665
$E_{2}$	12.39	$e_{24}$	0.0258
$E_{3}$	40.44	$e_{15}$	13.668
**Shear Modulus (GPa)**		**Dielectric Relative Constants**	
$G_{12}$	6.03	$\varepsilon_{11}^{T}/\varepsilon_{0}$	1574.8
$G_{23}$	6.68	$\varepsilon_{22}^{T}/\varepsilon_{0}$	24.7
$G_{31}$	17.01	$\varepsilon_{33}^{T}/\varepsilon_{0}$	1528.7
**Poisson’s Ratio**			
$\nu_{12}$	0.39		
$\nu_{23}$	0.17		
$\nu_{13}$	0.44		

## Data Availability

The data presented in this study are contained within the article. No additional data were created or analyzed in this study.

## Abbreviations

The following abbreviations are used in this manuscript:

PEH	Piezoelectric Energy Harvester
TO	Topology Optimization

## Appendix A

The generalized eigenvalue of a Matrix pencil (K,M) is defined by:

(A1) $$K\phi_{i}=\lambda_{i}M\phi_{i}\Leftrightarrow \left(K-\lambda_{i}M\right)\phi_{i}=0.$$

Therefore, the derivative w.r.t to $\rho_{e}$ can be computed by:

(A2) $$\frac{\partial (K-\lambda_{i}M)}{\partial \rho_{e}}\phi_{i}+\left(K-\lambda_{i}M\right)\frac{\partial \phi_{i}}{\partial \rho_{e}}=0.$$

By multiplying with $\phi_{i}^{T}$ from the left, we can eliminate the second part of the equation:

(A3) $$\begin{array}{cc} \phi_{i}^{T}\frac{\partial (K-\lambda_{i}M)}{\partial \rho_{e}}\phi_{i} & =0 \end{array}$$

(A4) $$\begin{array}{cc} \phi_{i}^{T}\frac{\partial K}{\partial \rho_{e}}\phi_{i}-\phi_{i}^{T}\frac{\partial \lambda_{i}}{\partial \rho_{e}}M\phi_{i}-\phi_{i}^{T}\lambda_{i}\frac{\partial M}{\partial \rho_{e}}\phi_{i} & =0. \end{array}$$

As $\phi_{i}^{T}M\phi_{i}=1$ by normalization, it follows:

(A5) $$\frac{\partial \lambda_{i}}{\partial \rho_{e}}=\phi_{i,e}^{T}\left(\frac{\partial K_{e}}{\partial \rho_{e}}-\lambda_{i}\frac{\partial M_{e}}{\partial \rho_{e}}\right)\phi_{i,e}.$$

## Appendix B

The derivative of $p_{el}$ can be computed as:

(A6) $$\frac{\partial p_{el}}{\partial \rho_{e}}=\frac{\partial }{\partial \rho_{e}}\frac{1}{2}\phi_{el}^{T}K_{el}\phi_{el}=\frac{1}{2}\left(\phi_{el}^{T}K_{el}\frac{\partial \phi_{el}}{\partial \rho_{e}}+\frac{1}{2}\phi_{el}^{T}\frac{\partial K_{el}}{\partial \rho_{e}}\phi_{el}\right).$$

$\frac{\partial K_{el}}{\partial \rho_{e}}=0$ since dielectric properties do not change with the change of design space. $\frac{\partial \phi_{el}}{\partial \rho_{e}}$ can be computed using the adjoint method. The harmonic equilibrium equation from the System (3) for an arbitrary excitation frequency *f* reads:

(A7) $$\underset{=:\bar{K}}{\underset{︸}{\left[\begin{array}{cc} K_{m}-\lambda M_{m} & K_{m,el}\\ K_{m,el}^{T} & K_{el} \end{array}\right]}}\underset{=:\phi }{\underset{︸}{\left[\begin{array}{c} \phi_{m}\\ \phi_{el} \end{array}\right]}}=\left[\begin{array}{c} Bu\\ 0 \end{array}\right],$$

with $\lambda ={\left(2\pi f\right)}^{2}$. Its derivative w.r.t $\rho_{e}$ is provided by:

(A8) $$\frac{\partial \bar{K}}{\partial \rho_{e}}\phi +\bar{K}\frac{\partial \phi }{\partial \rho_{e}}=0\Leftrightarrow \frac{\partial \phi }{\partial \rho_{e}}={\bar{K}}^{-1}\frac{\partial \bar{K}}{\partial \rho_{e}}\phi .$$

Now, let ${\left[\mu_{m},\mu_{el}\right]}^{T}$ solve the following adjoint system:

(A9) $$\bar{K}\underset{\mu }{\underset{︸}{\left[\begin{array}{c} \mu_{m}\\ \mu_{el} \end{array}\right]}}=\left[\begin{array}{c} 0\\ \frac{1}{2}K_{el}\phi_{el} \end{array}\right].$$

Combining (A8) with (A9) and using the symmetry of $\bar{K}$, (A6) can be rewritten as:

(A10) $$\frac{\partial p_{el}}{\partial \rho_{e}}=\mu^{T}\frac{\partial \bar{K}}{\partial \rho_{e}}\phi$$

From here, only:

(A11) $$\frac{\partial p_{el}}{\partial \rho_{e}}=\mu_{e}^{T}\left(\frac{\partial K_{e}}{\partial \rho_{e}}-\lambda \frac{\partial M_{e}}{\partial \rho_{e}}\right)\phi_{e}$$

remains, as the piezoelectric properties stored $K_{m,el}$ is also independent from $\rho_{e}$ and $\mu_{el,e}=0$ and $\phi_{el,e}=0$ for all the design space elements (steel structure).
